# The effect of neck-specific exercise with or without a behavioral approach on psychological factors in chronic whiplash-associated disorders: A randomized controlled trial with a 2-year follow-up

**DOI:** 10.1097/MD.0000000000004430

**Published:** 2016-08-26

**Authors:** Thomas Overmeer, Gunnel Peterson, Maria Landén Ludvigsson, Anneli Peolsson

**Affiliations:** a Physiotherapy Department, School of Health, Care and Social Welfare, Mälardalen University, Västerås; b Centre for Health and Medical Psychology, Örebro University, Örebro; c Department of Medical and Health Sciences, Division of Physiotherapy, Linköping University, Linköping; d Centre for Clinical Research Sörmland, Uppsala University, Uppsala; e Rehab Väst, County Council of Östergötland, Department of Rehabilitation and Department of Medical and Health Sciences, Linköping University, Sweden.

**Keywords:** behavioral medicine, exercise therapy, neck, spine, whiplash injuries

## Abstract

**Background:**

To investigate the effect of neck-specific exercise with (NSEB) or without (NSE) a behavioural approach and prescribed physical activity (PPA) on general pain disability and psychological factors in chronic whiplash-associated disorders (WAD), grade 2 and 3, with a 2-year follow-up.

**Methods:**

A randomized controlled multi-centre study of 3 exercise interventions (NSE, NSEB or PPA) including a 2-year follow-up. A total of 216 volunteers with chronic WAD were recruited and 194 were analyzed, mean age 40.4 (Standard Deviation [SD] 11.4). Measures of general pain disability, pain catastrophizing, anxiety and depression, and kinesiophobia were evaluated at baseline, and 3, 6, 12 and 24 months with linear mixed models.

**Results:**

General pain disability decreased by 28% in the NSEB group from baseline to 3 months (*P* < 0.001) and the improvements in disability were maintained over time (6, 12 and 24 months *P* < 0.01) compared to the NSE (*P* > 0.42) and PPA groups (*P* > 0.43). Pain catastrophizing decreased in the NSE group from baseline to 6 and 12 months (*P* < 0.01) and in the NSEB group from baseline to 3 and 24 months (*P* < 0.01) compared to the PPA group (*P* > 0.82) that showed no change over time. The NSE group improved in kinesiophobia over time from baseline to12 months (*P* < 0.01) compared to the NSEB (*P* = 0.052) and the PPA groups (*P* > 0.74). Anxiety decreased over time from baseline to 12 and 24 months in the NSE group (*P* > 0.02), but not in the NSEB (*P* > 0.25) or the PPA (*P* > 0.50) groups. The PPA had no effect on general disability or any of the measured psychological factors.

**Conclusion:**

This randomised controlled trial with a 2-year follow-up shows that physiotherapist-led neck-specific exercise with or without the addition of a behavioural approach had superior outcome on general disability and most psychological factors compared to the mere prescription of physical activity.

## 1 Introduction

Whiplash is an acceleration–deceleration mechanism of energy transfer to the neck and head from indirect neck trauma. Symptoms following whiplash trauma can be for example, neck pain, headache, dizziness, and cognitive symptoms, leading to disability, illness, and reduced work ability.^[1,2]^ The cumulative incidence of patients seeking health care for whiplash-associated disorders (WADs) has been estimated to >3/1000 inhabitants in North America and Western Europe.^[1]^ Patients remaining symptomatic or showing residual disability after 6 months are considered to have chronic WAD^[3]^ and is a challenging condition for clinicians.^[1]^ Similar to other chronic pain conditions, central pain processing is likely to play a crucial role in the transition from acute toward chronic WAD.^[4]^ Consistent with other chronic pain conditions,^[6]^ a number of psychological factors, together with high initial pain and disability, have been found to play a role in the central pain processing in WAD.^[5–13]^ Pain-related catastrophizing, broadly defined as an exaggerated negative orientation toward pain stimuli and pain experience,^[14]^ has shown to lead to a 3.8 times greater risk for chronicity^[5]^ and was associated with lower perceived health and quality of life.^[6]^ Pain-related catastrophizing is believed to play an important role in the complaints of patients with chronic WAD when referred to a physical therapist.^[7]^ Fear avoidance was found to be predictive for the development of chronic WAD,^[13]^ and it is advocated to be a promising model for understanding the development of persistent complaints after an acute whiplash injury.^[15]^ Depression also appears to predict WAD recovery,^[8]^ and persistent depression is seen in patients 5 years after a whiplash injury.^[9]^ In order to adequately address these psychological factors, cognitive behavioral components in physiotherapy management of chronic WAD have been recommended.^[4,16,17]^ Moreover, based on the available research, exercise programs are suggested to be the most effective noninvasive treatment for patients with chronic WAD.^[18,19]^ Commonly recommended in Sweden is to advise WAD patients to perform general physical activity.

Pain intensity and neck-specific disability have earlier shown to be superiorly treated with neck-specific exercises (NSEs) alone or combined with behavioral components compared with prescribed general physical activity.^[19]^ However, if this is true also for patients’ self-rated general disability from pain and psychological factors is unknown.

The aim of the study was to investigate the effect of NSE with or without a behavioral approach and prescribed physical activity (PPA) on general pain disability and psychological factors in chronic WADs, grades 2 and 3, with a 2-year follow-up.

## 2 Methods

### 2.1 Overview

This study was a planned analysis of secondary outcomes of a randomized controlled multicenter study with assessor and group allocation blinding including a 2-year follow-up. Participants were randomized to 1 of 3 alternative physiotherapy treatments: NSE followed by PPA, NSE with a behavioral approach (NSEB) followed by PPA, and PPA by a physiotherapist without NSE. The Regional Ethical Committee at Linköping University, Sweden, approved the study.

### 2.2 Setting and participants

**Figure 1:**
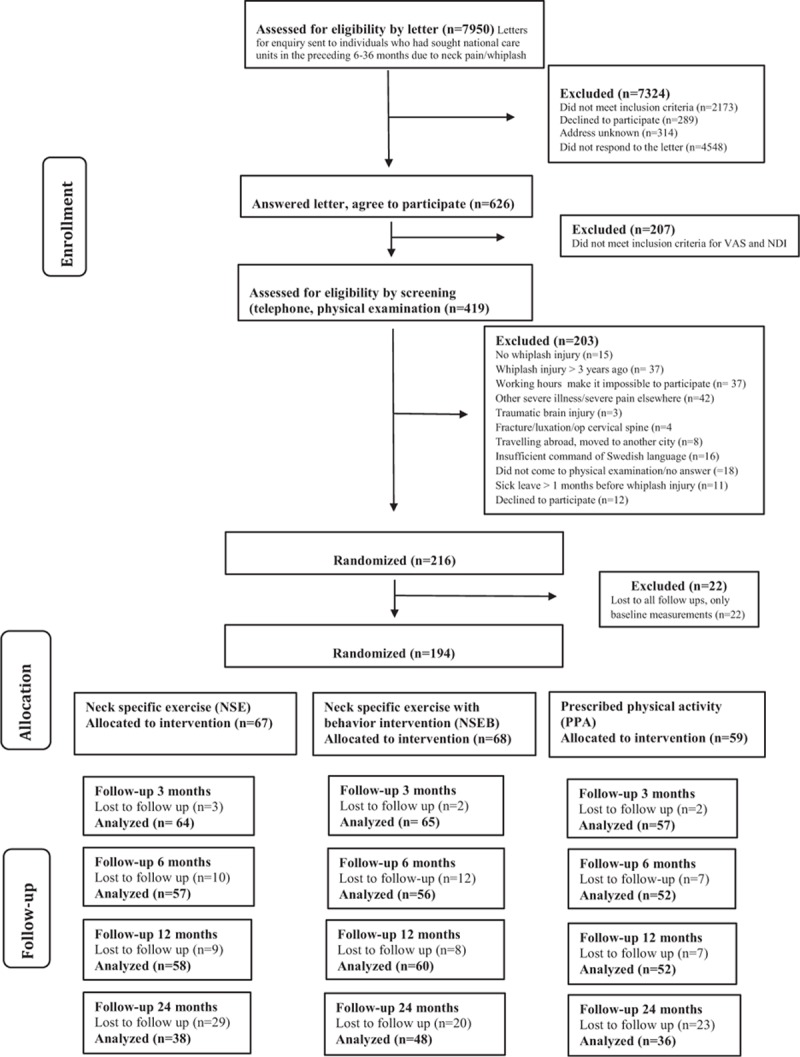
Flowchart of the participants through the study.

In accordance with the study protocol (ClinicalTrials.gov identifier: NCT01528579),^[20]^ participants were individuals with chronic WAD grade 2 or 3.^[3]^ A total of 216 participants were included and 194 were analyzed. Inclusion criteria were age between 18 and 63 years, WAD grades 2 to 3 after a whiplash injury at least 6 months but not more than 3 years ago, pain intensity >20 mm on a 100-mm Visual Analog Scale, and/or >20% on the Neck Disability Index (NDI, 0%–100%). Exclusion criteria were known or suspected serious physical pathology, including myelopathy, spinal tumor, spinal infection, or ongoing malignancy, earlier fracture or luxation of the cervical column; neck trauma with persistent symptoms from previous injury, surgery in the cervical column, neck pain that caused >1-month absence from work in the year before the whiplash incident, signs of traumatic brain injury at the time of the trauma (unconsciousness, retrograde or posttraumatic amnesia, disorientation, or confusion), generalized or a more dominant pain elsewhere in the body, diseases or other injuries that might prevent full participation in the study, diagnosis of a severe psychiatric disorder, known drug abuse, insufficient knowledge of the Swedish language (inability to answer the questionnaires). For a detailed description of the participants, see Table 1 and Fig. 1 for the flowchart.

**Table 1:**
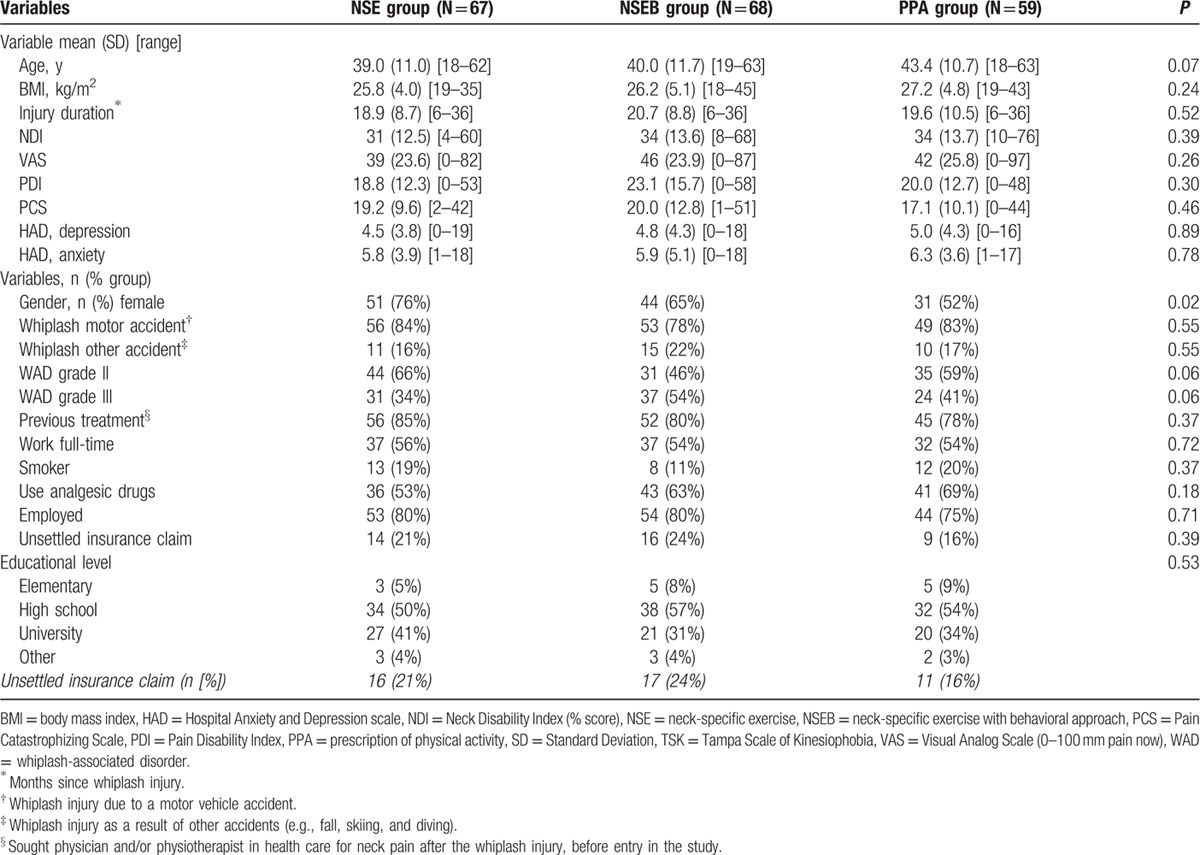
Baseline characteristics.

Several participants reported use of analgesic drugs due to neck pain at baseline. This was distributed over the groups as follows: use of analgesic drugs yes/no (%), NSE 40/35 (53/47), NSEB 44/27 (62/38), and PPA 45/22 (67/33).

The vast majority of the patients were born in Sweden (n = 186), 13 were born in another Nordic country, 5 in the rest of Europe, and 12 were born outside Europe.

Physiotherapists in primary care centers or private outpatient clinics in 6 different counties in Sweden performed the treatment. They were experienced in managing patients with neck pain disorders, and the treatment was conducted within the publicly funded reimbursement system. The physiotherapists participated in a 1-day educational session and practiced the different interventions.

### 2.3 Procedure

Healthcare registers of 6 Swedish counties, including primary health care, specialist orthopedic clinics, and hospital outpatient services, were examined for potential participants. Participants were screened for eligibility by a 4-step process. A detailed description of the procedure is described in earlier articles on this trial.^[19,21]^ Participants were recruited between February 2011 and May 2012, and all participants received written and verbal information about the study. Informed consent and baseline data were collected before allocation.

### 2.4 Randomization and masking

An independent researcher made a computerized randomization list and put the individual results in sealed completely opaque envelopes to be distributed to the treating physiotherapists. The study was assessor and group allocation blinded. Due to the nature of the intervention, blinding of the participants and physiotherapists was not possible.

### 2.5 Interventions

The 3 interventions were physiotherapist-led NSE, NSE with the addition of an NSEB, or PPA. All 3 interventions were provided by physiotherapists in primary care and were undertaken over a 12-week period. In the NSE group, participants received supervised NSE twice weekly. Progressive resistance training was introduced in the gym with a focus on low-load endurance training. A detailed description of the exercises can be found at the Academic Archive On-line.^[19]^ Exercise pain provocation was avoided in this group. Toward the end of the 12-week exercise period, participants were encouraged to engage in general physical activity and to continue NSE at home. In the NSEB group, the exercise protocol was the same as that undertaken by the NSE group, but with the addition of a behavioral approach. According to the concept of graded exercise, patients were encouraged not to focus on temporary increases in neck pain.^[22]^ They also received behavioral interventions including education and introduction to activities aimed at pain management and problem-solving, guided by a physiotherapist. Participants in the PPA group initially received a physical examination and a short motivational interview conducted by a physiotherapist. They were then prescribed individualized physical activity to be performed independently based on the interview and the physical examination. NSEs including any form of head resistance were not prescribed in this group. Further details about the intervention protocols have been published previously.^[19]^

### 2.6 Outcomes and follow-up

All outcome measures were self-administered and filled in by the patients at their homes. The Pain Disability Index (PDI) and the Pain Catastrophizing Scale (PCS) were collected at baseline, and 3, 6, 12, and 24 months after inclusion. The Hospital Anxiety and Depression Scale (HAD) and the Tampa Scale of Kinesiophobia (TSK) short version were collected at baseline, 6, 12, and 24 months after inclusion.

#### 2.6.1 Primary outcome measure

The PDI was used to measure domain-specific and general disability related to chronic pain.^[23]^ It is a 7-item inventory designed to measure the degree to which pain interferes with functioning across a range of activities. Each item score can range from 0 (no interference) to 10 (total inference). Thus, the total PDI score can range from 0 to 70. The PDI has shown good reliability and validity.^[23–25]^

#### 2.6.2 Secondary outcome measures

The PCS was used to measure the extent of pain catastrophizing in participants.^[14]^ The PCS is a 13-item scale in which participants are asked to reflect on past painful experiences and indicate the degree to which they experienced thoughts or feelings during pain on a 5-point scale, ranging from 0 (not at all) to 4 (always). The scale has been shown to have good reliability and validity in pain outpatient samples.^[26,27]^

The HAD was used to measure anxiety and depression.^[28]^ HAD is a 14-item scale with 2 subscales—1 subscale of 7 items for anxiety and 1 subscale of 7 items for depression. It was designed to measure both anxiety and depression. It has a 4-point scale for each question, ranging from 0 (not at all) to 3 (very often). It has shown to be a valid clinical indicator of the possibility of depression and clinical anxiety in a Swedish population.^[29]^

The TSK short version (TSK-11) was used to measure fear of movement and (re)injury. The 11-item scale (score range 11–44) is a short version of the original TSK. It evaluates fear of movement with higher scores indicating greater fear of movement, and each item is scored on a 4-point scale. Scoring possibilities range from “strongly disagree” (score = 1) to “strongly agree” (score = 4). The TSK-11 possesses similar psychometric properties to the original TSK.^[30]^

### 2.7 Statistical analysis

The sample size for the whole randomized controlled trial was based on the primary outcome NDI.^[19]^ A total of 216 patients were recruited. One hundred ninety four patients were analyzed using linear mixed models. This method requires at least 2 measures besides baseline. Data were analyzed by using SPSS (IBM SPSS, Statistics for windows, Version 22.0, Armonk, NY). All participants, who completed each measurement, were included in the analysis on an intention-to-treat basis. An α level of <0.05 was used for statistical significance. Background data were analyzed with one-way analyses of variance or the Kruskal–Wallis test for nonnormally distributed data. The chi-square test was used for binary outcomes.

Pain disability (PDI), pain catastrophizing (PCS), anxiety and depression (HAD), and kinesiophobia (TSK-11) were analyzed with linear mixed model.^[31]^ For all models, exercise group (3 levels; NSE, NSEB, and PPA), time (5 levels for PDI and PCS; baseline, 3, 6, 12, and 24 months, and 4 levels for HAD and TSK; baseline, 6, 12, and 24 months), gender (2 levels; men and women), and the interaction between group and time were included as fixed factors. The linear mixed model *P* values were reported for the within-group differences over time (Pt); differences between groups, estimated marginal means (Pg); differences between sex (Ps); interaction between time and group; if significant means that one or more of the groups are changing over time but the groups are changing in different ways (Pt × g) and interaction between time, group, and sex (Pt × g × s).

### 2.8 Funding

This study was supported by funding from the Swedish government through the REHSAM Foundation (RS2010/009), the Swedish Research Council (521-2014-2982), the regional Center for Clinical Research and the County Council of Östergötland, Centre for Clinical Research Sörmland at Uppsala University, the Medical Research Council of Southeast Sweden, and the Uppsala-Örebro Regional Research Council.

## 3 Results

One hundred ninety four individuals with chronic WAD were analyzed in this study, 126 women and 68 men, with a mean age of 40.4 years (Standard Deviation [SD] 11.4). There were no differences between groups at baseline except for significantly more women and younger participants in the NSE group compared to the PPA group (Table 1). A total of 194 participants were included in the linear mixed model for PDI and PCS and 179 participants in HAD and TSK. Participants lost to follow-up did not significantly differ in baseline measurements (PDI, PCS, HAD, or TSK) (*P* > 29) or baseline characteristics (*P* > 0.10), except for visits to physician. Dropouts had sought physicians significantly fewer times (mean 0.33; SD 0.48) compared to those included in the mixed model analyses (mean 0.67; SD 0.47, *P* < 0.01). Tables 2 and 3 display the mean, standard error, and degrees of freedom for all groups and all measures at all time points.

**Table 2:**
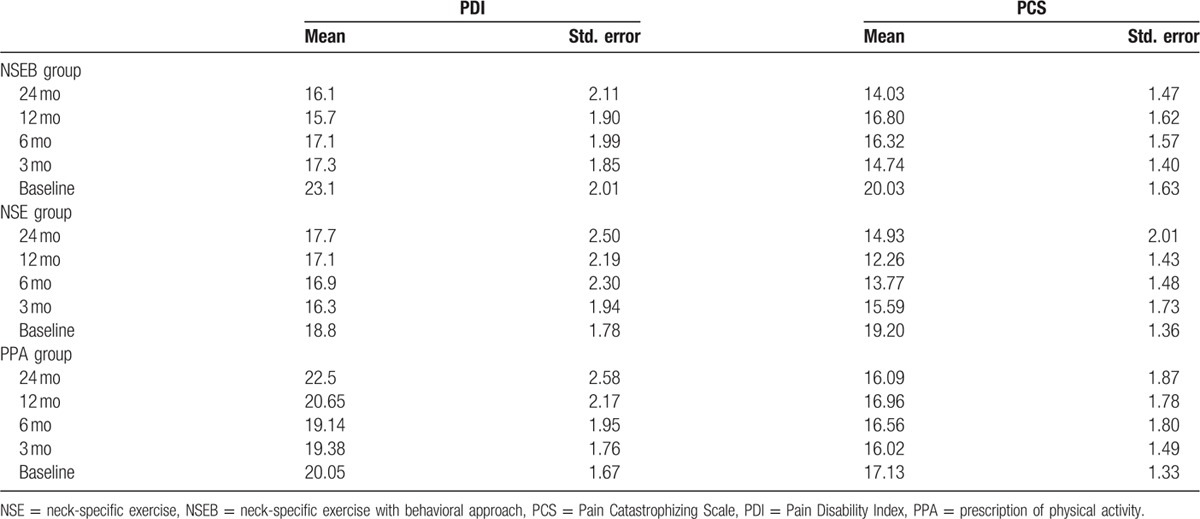
Mean, standard error for the PDI and PCS for all groups at all time points.

**Table 3:**
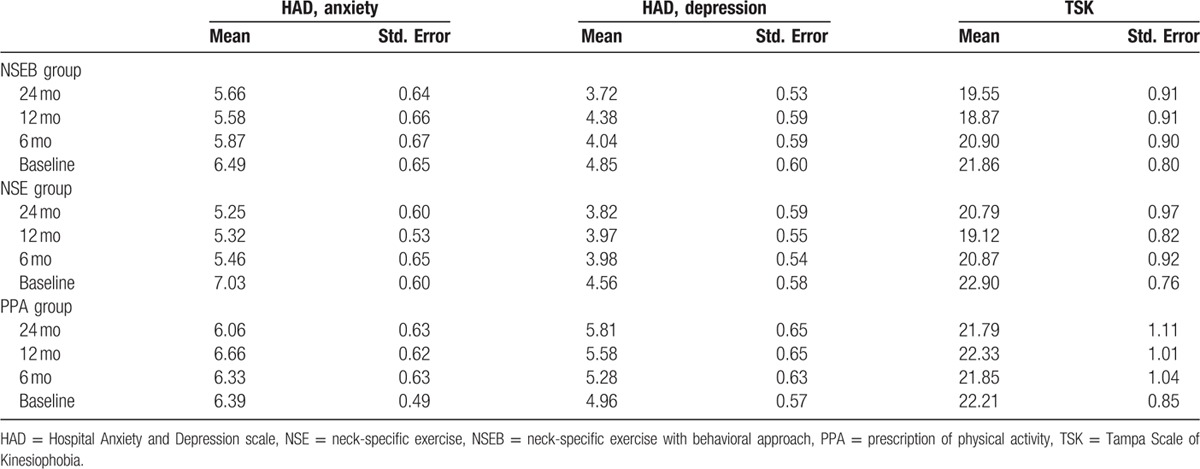
Mean, standard error for the HAD and TSK for all groups at all time points.

### 3.1 Pain disability

There was a significant change over time effect (F = 4.1, *P* < 0.01) and main group by time interaction effect (F = 2.0, *P* < 0.05). Pain disability decreased in the NSEB group from baseline to 3 months, and the improvements in disability were maintained over time (6, 12, and 24 months F = 6.3, *P* < 0.01) compared to the NSE and PPA groups. There was no change over time in the NSE (F = 1.0, *P* > 0.42) or the PPA groups (F = 1.0, *P* > 0.43). There were no gender differences (F = 0.28, *P* > 0.60) or interaction between group, time, and sex (F = 1.2, *P* < 0.31). For a detailed description of the results, see Table 4.

**Table 4:**
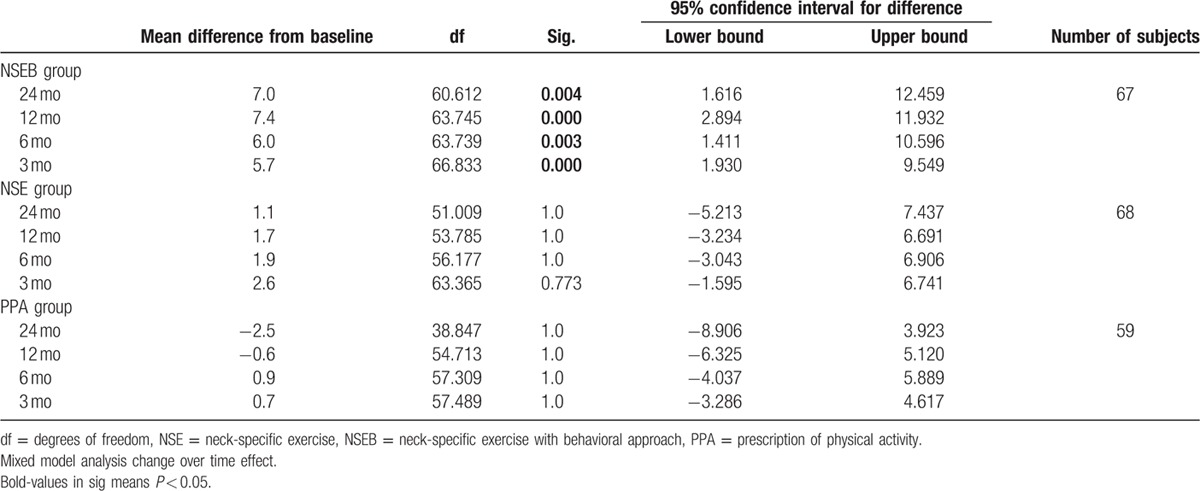
Pain disability measured by the PDI.

### 3.2 Pain catastrophizing

There was a significant change over time effect (F = 6.7, *P* < 0.01) and main group by time interaction effect (F = 2.3, *P* > 0.03) in pain catastrophizing. Pain catastrophizing decreased in the NSE group from baseline to 6 and 12 months (F = 6.9, *P* < 0.01) and in the NSEB group from baseline to 3 and 24 months (F = 6.3, *P* < 0.01) compared to the PPA group (F = 0.38, *P* > 0.82) that showed no change over time. There were no group differences between gender (F = 2.1, *P* = 0.15) or interaction between group, time, and sex (F = 0.78, *P* < 0.62). For a detailed description of the results, see Table 5.

**Table 5:**
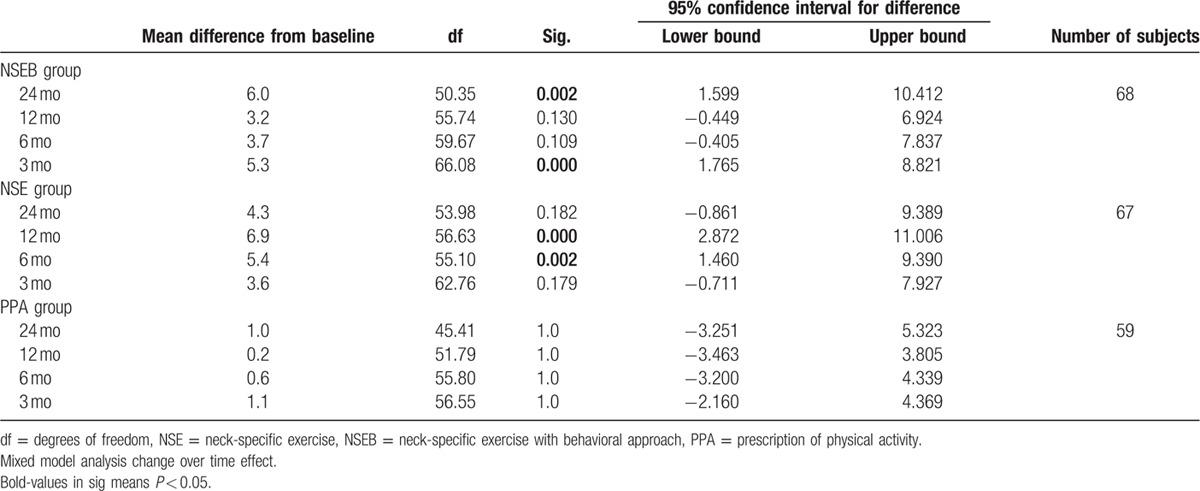
Pain catastrophizing measured by the PCS.

### 3.3 Anxiety and depression

Regarding anxiety, there was a significant change over time effect (F = 3.9, *P* = 0.01) but no significant group by time interaction effect (F = 1.6, *P* > 0.13). Anxiety decreased over time from baseline to 12 and 24 months in the NSE group (F = 4.5, *P* > 0.02), but not in the NSEB (F = 1.4, *P* > 0.25) or the PPA (F = 0.80, *P* > 0.50) groups. There were no gender differences (F = 0.2, *P* > 0.62) or interaction between group, time, and sex (F = 0.28, *P* > 0.94).

Regarding depression, there was no significant change over time effect (F = 0.96, *P* = 0.41), group by time interaction effect (F = 1.6, *P* = 0.16), differences between groups (F = 1.8, *P* > 0.16), gender differences (F = 0.57, *P* = 0.45), or interaction between group, time, and sex (F = 1.0, *P* > 0.38). For a detailed description of the results, see Tables 6 and 7.

**Table 6:**
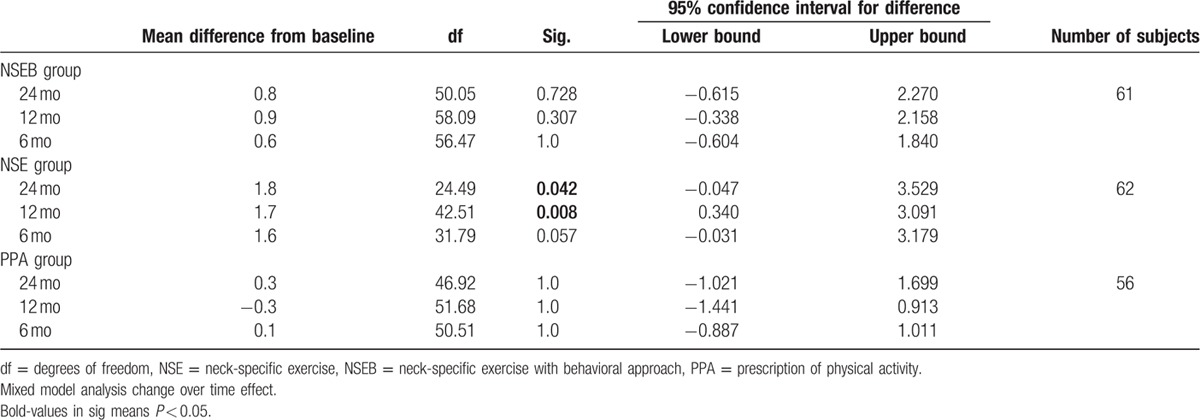
Anxiety measured by the HAD.

**Table 7:**
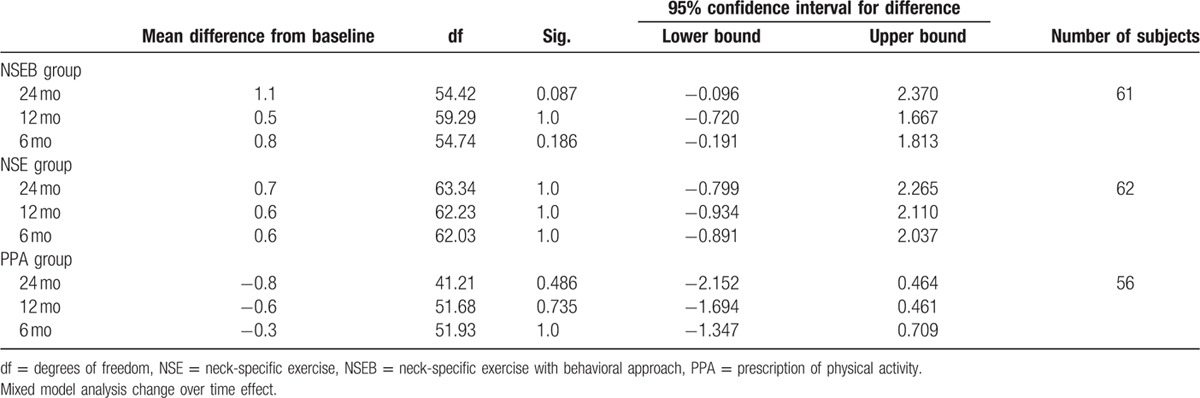
Depression measured by the HAD.

### 3.4 Kinesiophobia

There was a significant change over time effect (F = 5.9, *P* < 0.01) and main group by time interaction effect (F = 2.9, *P* = 0.01) in kinesiophobia. The NSE group improved over time from baseline to12 months (F = 7.6, *P* < 0.01) compared to the NSEB (F = 2.7, *P* = 0.052) and the PPA groups (F = 0.42, *P* > 0.74).

There was a significant effect of gender. In the NSE group, men showed significant higher kinesiophobia at baseline and 6 months compared to women in the NSE group (F = 4.7–4.6, *P* < 0.04) and in the PPA group at 6 months (F = 4.1, *P* < 0.5). They were also close to significant gender difference in the NSEB group at baseline (F = 3.9, *P* > 0.054) where men had tendency to higher kinesiophobia. For a detailed description of the results, see Table 8.

**Table 8:**
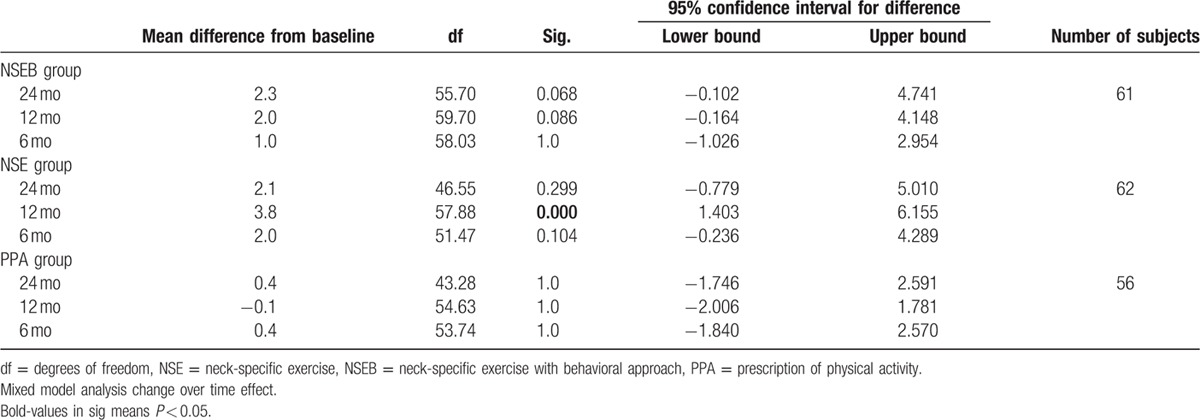
Kinesiophobia measured by the TSK-11.

## 4 Discussion

The results of this randomized controlled trial with a 2-year follow-up show that physiotherapist-led NSEB or NSE had better outcome on general disability and most psychological factors compared to the mere PPA. NSE improved in 3 out of 5 variables; catastrophizing, kinesiophobia, and anxiety. NSEB improved in PDI and catastrophizing and PPA in none of the variables. More specifically, neck-specific training with the addition of a behavioral component significantly decreased general pain disability by 28% in the first 3 months and sustained this over 2 years. The active physiotherapy-led treatment, with or without the behavioral component, also decreased pain catastrophizing, while PPA did not. Moreover, the NSE group significantly diminished kinesiophobia the first year and anxiety over 2 years, while there was a trend toward lower kinesiophobia in NSEB group. NSEs with the behavioral component also showed a trend toward lower depression. The PPA had no effect on general disability or any of the measured psychological factors. The fact that active treatment by means of neck-specific training has superior effect on chronic WAD patients compared to the mere PPA has been reported before for pain and neck-specific disability.^[19]^ This trial now demonstrates similar results for general disability and several psychological factors and shows these results to be stable over time.

However, the effect in this 2-year follow-up was modest and only on disability a decisive difference could be seen in the outcome between the physiotherapist-led NSE leg and the intervention with the addition of a behavioral approach. This may not be so surprising because our behavioral approach was based on Cognitive Behavioural Therapy (CBT) principles, and a recent review of CBT treatment on chronic pain patients^[32]^ concluded that CBT in addition to another interventions did not result in significantly better results. Moreover, the review showed only evidence for a short-time effect. In the present study, the effects were sustained over a longer period of time. Yet, there may be several additional explanations for the outcome.

A first reason for modest difference in the outcome between the physiotherapist-led NSE leg and the intervention with the addition of a behavioral approach on psychological factors may lay in the study population. In a recent study, Soer et al^[33]^ calculated a reference value for general disability, measured by the PDI, for WAD patients (mainly from multispecialty tertiary care centers) to a mean of 37.6 points. The patients in the different treatment groups in our study had 18.8 to 23.1 as means, indicating considerably lower disability than Soers’ WAD patients at baseline. Moreover, if we compare the means of our patients (WAD grades 2 and 3) concerning pain catastrophizing with Sullivan et al,^[34]^ it shows that the patients in Sullivan's study of WAD patients (grades 1 and 2) had a mean of 27 points on the PCS, while our groups scored 17.1 to 19.2 at baseline, indicating again a considerably lower score for the patients in the present study. If we continue to look at anxiety and depression, our participants presented a mean score of 4.5 to 6.3 on HAD, which is below the cutoff score of 8 for low mood or low anxiety.^[35]^ Taken together, there are clear indications that the present study population at baseline had low disability, pain catastrophizing, depression, and anxiety. This leaves less room for improvement compared to patients from rehabilitation or pain clinics with higher baseline scores.

Furthermore, more than 50% of the patients in the present study worked full-time and around 80% had employment at treatment start, suggesting a relatively high percentage of adaptive coopers. Adaptive coopers are patients dealing well with the psychological and emotional problems incorporated in chronic pain and characterized by lower interference with everyday activities, lower affective distress, and higher life control.^[36–38]^ For adaptive coopers, there is little room for improvement, and the behavioral approach might have had little impact on outcome contributing to the similar results for psychological factors for both active treatment arms. In concordance with this, Sullivan et al^[34]^ showed in a study of chronic WAD patients using a psychosocial risk-factor-targeting intervention that this treatment was only successful for patients displaying many psychosocial factors, whilst the intervention was superfluous for patients without these factors. So the patients in our study with a low degree of psychological factors that were randomized to the behavioral treatment arm might not have benefited much from the addition of the behavioral component. Yet, a 28% reduction in general disability was accomplished with the active treatment incorporating the behavioral component and was sustained over a 2-year period. The Minimal clinical important change (MCIC) for the PDI scale for chronic back pain patients, including neck pain patients, has been calculated to be 8.5 to 9.5 points.^[39]^ This was calculated from a mean of 34.6 corresponding to a 26% reduction. Participants in our study reduced their disability with 6 points but from a substantially lower starting point, 23.1 to 18.8 points, resulting in a similar MCIC of 26% reduction on general disability.

Participants in our study were identified through healthcare registers of 6 Swedish counties, including primary health care, specialist orthopedic clinics, and hospital outpatient services. They were contacted by mail and invited to participate in a study for WAD patients that included a form of exercise. Given the low baseline scores on disability, pain catastrophizing, anxiety, and depression in comparison with other studies, the data suggest that we might have encountered a selection bias. It seems reasonable to assume that patients with lower levels of distress and other psychological discomfort would be more inclined to submitting themselves to training and therefore probably chose to participate, while those with more prominent psychological factors may have chosen not to respond to the invitation.

The second reason for modest difference in the outcome may have to do with the patients’ characteristics in combination with treatment allocation. In our study, patients were allocated to the treatment arms irrespectively of their characteristics. Patients receiving the specific neck exercises without the behavioral approach were treated according to a pain contingent approach, and exercise pain provocation was avoided in this group. In accordance with the concept of graded exercise, patients who received the behavioral approach were encouraged not to focus on temporary increases in neck pain and were treated according to a time contingent regime. This may have increased the risk of augmenting nociceptive barrage toward the central nervous system during and following treatment in patients with signs of central sensitization, which in turn is likely to sustain the process of central sensitization. Nijs et al^[4]^ advocated in his review to take central sensitization into account when prescribing exercise and to avoid time contingent exercise for patients with signs of central sensitization. This would mean that time contingent behavioral approach would be suitable for patients without sensitization and exercise with pain contingent approach would be more suitable for patients with signs of sensitization. Because our patients were randomly allocated to the different treatment arms instead of allocated based on sign of sensitization, and assuming that patients with central sensitization were equally distributed between the active treatment arms, the effect of the intervention may have been partially washed out.

Great care was taken into recruiting the physiotherapists administering the behavioral treatment. The vast majority had an interest in behavioral medicine and many had attended postgraduate courses in this field before participating in the study. They all received an additional 1-day training on how to deliver the behavioral treatment before treatment start. Although 2 recent systematic reviews^[40,41]^ show that physiotherapists working with pain patients only partially recognized cognitive, psychological, and social factors, we have no reason to believe that this played a major role in the outcome. Treatment with a behavioral approach has been advocated^[42]^ and tried before,^[17]^ but to our knowledge, the present study is the only study that shows a positive outcome of a physiotherapist-led behavioral approach on chronic WAD patients grades 2 and 3.

The strength of this study is that it has a 2-year follow-up, which enables us to investigate if the results are stable and sustained over time. It also has a relatively large number of participants, which makes the findings robust. It includes individuals with WAD grade 3, often excluded in other studies and was assessor blinded and conducted at multiple primary care centers. Although the study had a strict treatment protocol, a weakness of this study is that we do not have any data on actual practice behavior and therefore cannot be completely certain that the treatment was given in the way it was intended. Although all participating physiotherapists were experienced in managing patients with neck pain disorders, we cannot be sure of the specific competency of the physiotherapists in delivering a behavioral approach. Another limitation was the lack of no-intervention control group. However recently, Peolsson et al^[43]^ using the same NSE treatment as in the present study showed NSE to be superior to staying on a waiting list.

For future research, given these modest results and the uncertainty of the impact of the possible other explanations, maybe a new line of research should be opened. Instead of submitting all patients irrespective of their characteristics to the same kind of treatment, like in traditional randomized controlled trials, tailoring the treatment to patient characteristics could be a more successful approach. In this kind of study, both central sensitization and potential psychosocial barriers for recovery could be taken into account as potential modifiers. A behavioral approach can consist of different components. It should include oral education regarding physiological and psychological aspects of pain^[44]^ as well as challenging and changing inappropriate pain beliefs.^[45]^ It is often administered in a time contingent fashion. A new cognitive approach addressing the educational and cognitive aspect but administered on a pain contingent basis might be more appropriate for patients with signs of central sensitization and psychosocial risk factors. A behavioral approach on a time contingent basis is probably more appropriate for patients without signs of central sensitization but with potential psychosocial barriers for recovery. Instead of a one-size-fits-all strategy that ignores the heterogeneity in chronic WAD patients, predetermined patient characteristics will decide to what type of intervention they will be assigned, with a nonstratified group as control for the results. This type of stratified approach has been successfully tested before on other pain patients and in physiotherapy settings.^[46,47]^

In summary, the results of this randomized controlled trial with a 2-year follow-up show that physiotherapist-led NSEB or NSE had better outcome on general disability and most psychological factors compared to the mere PPA. The PPA had no effect on general disability or any of the measured psychological factors. More specifically, neck-specific training with the addition of a behavioral component significantly decreased general pain disability by 26% in the first 3 months and sustained this over 2 years. The active physiotherapy-led treatment with or without the behavioral component also decreased pain catastrophizing, while PPA did not. Moreover, NSEs significantly diminished kinesiophobia the first year and anxiety over 2 years. To our knowledge, the present study is the only study that shows a positive outcome of a physiotherapist-led behavioral approach on chronic WAD patients grades 2 and 3.
